# Prognostic Significance of MicroRNA-375 Downregulation in Solid Tumors: A Meta-Analysis

**DOI:** 10.1155/2014/626185

**Published:** 2014-10-23

**Authors:** Yingjie Shao, Yiting Geng, Wendong Gu, Jin Huang, Zhonghua Ning, Honglei Pei

**Affiliations:** ^1^Department of Radiation Oncology, The Third Affiliated Hospital of Soochow University, 185 Juqian Street, Changzhou, Jiangsu Province 213003, China; ^2^Department of Oncology, The Third Affiliated Hospital of Soochow University, 185 Juqian Street, Changzhou 213003, China

## Abstract

*Objective*. Recently, many studies have shown that microRNAs (miRNA) exhibit altered expression in various cancers and may play an important role as prognostic biomarker of cancers. We performed a meta-analysis to evaluate the impact of miR-375 expression in solid tumors on patients' overall survival (OS). *Methods*. Studies were identified by searching PubMed, Embace, and Cochrane Library (last search update was in May 2014) and were assessed by further quality evaluation. The pooled hazard ratios (HRs) with 95% confidence intervals (CIs) for total and stratified analyses were calculated to investigate the association between miR-375 expression and cancer patients OS. *Results*. Our analysis results indicated that downregulation of miR-375 predicted poor OS (HR = 1.91, 95% CI 1.48–2.45, *P* < 0.001). Subgroup analyses showed that lower expression of miR-375 was significantly related with poor OS in patients with esophageal carcinoma (HR = 2.24, 95% CI 1.69–2.96, *P* < 0.001) and non-small-cell lung cancer (NSCLC) (HR = 1.71, 95% CI 1.31–2.24, *P* < 0.001). *Conclusions*. The findings from this meta-analysis suggest that miR-375 expression is associated with OS of patients with malignant tumors and could be a useful clinical prognostic biomarker.

## 1. Introduction

Cancer is one of the most common causes of death worldwide and has become a major public health issue [1]. Hence, newer cancer biomarkers with high sensitivity and specificity are essential for the proper detection, treatment, and prognosis of this fatal disease. MicroRNAs (miRNAs), small noncoding RNAs with a length of approximate 22 nucleotides, have been demonstrated to have high potential prognostic value in many cancers. MiRNAs biogenesis begins with transcription of primary transcripts (pri-miRNAs) in cell nucleus. Catalyzed by the ribonuclease Drosha and its essential cofactor DGCR8, pri-miRNAs release a 60–80-nucleotide precursor (pre-miRNA) which is exported to cell cytoplasm and cleaved by Dicer to generate a 22-nucleotide duplex. One strand of the duplex is incorporated into the RNA-induced silencing complex (RISC) by bounding with Argonaute, whereas the other is degraded. Base-pairing between the miRNAs and target mRNAs (usually in the 3′ untranslated region) guides RISC to complementary transcripts, leading to translation repression or the target mRNAs degradation [2]. It is estimated that miRNAs regulate 1/3 to 2/3 of human genes [3]. Thus, the dysregulation of the biogenesis and function of miRNAs is often associated with human diseases, especially malignancies [4]. Accumulating evidence has demonstrated that miRNAs act as oncogenes or tumor suppressors by targeting genes involved in cell differentiation, proliferation, survival, apoptosis, and metastasis (reviewed in [5]). The expression of numerous miRNAs is dysregulated in various cancers, which is often associated with diagnosis, staging, progression, prognosis, and response to clinical therapies (reviewed in [6]).

MicroRNA-375 (miR-375) was originally identified from murine pancreatic *β*-cell line MIN6 as a pancreatic islet-specific miRNA [7]. The miR-375 gene is located in an intergenic region between the beta-A2 crystallin (cryba2) and undescribed coiled-coil domain-containing protein 108 (ccdc108) genes in human chromosome 2q35 region [8], a genomic region conserving the synteny between humans and mice. Moreover, the sequences of pre-miR-375 in both species present a 100% homology, highlighting the high degree of conservation for this specific miRNA [8]. Further study revealed that miR-375 is a multifunctional miRNA participating in pancreatic islet development, glucose homeostasis, mucosal immunity, lung surfactant secretion, and more importantly tumorigenesis (reviewed in [9]). Recently, miR-375 has been found significantly dysregulated in many human cancers [10–15]. In addition, miR-375 has been found to have prognostic value in a variety of tumors. Multiple studies reported a significant association between low miR-375 and poor prognosis [12, 16–25]. However, insignificant or even opposite results have been found in some other studies [26–30]. According to the evidence we have gained so far, it is still not enough to come to a conclusion whether miR-375 could be used as a potent biomarker for prognosis. Therefore, a systematical and comprehensive meta-analysis was carried out to investigate the relationship between miR-375 expression and the survival of patients with cancer. To our knowledge, this meta-analysis is the first to evaluate the prognostic value of miR-375 in cancer patients.

## 2. Materials and Methods

This meta-analysis was carried out in accordance with the guidelines of meta-analysis of observational studies in epidemiology (MOOSE) [31].

### 2.1. Search Strategy

Studies were performed by searching PubMed, Embace, and Cochrane Library (updated by May 2014). The search strategy was “microRNA-375 OR miR-375” AND “tumor OR neoplasm OR cancer OR carcinoma.” No language limitation was applied. The reference lists of relative articles were also screened to further identify potential studies. The comprehensive database search was carried out independently by two authors (Y. Shao and Y. Geng). The disagreements were resolved by consensus.

### 2.2. Inclusion and Exclusion Criteria

Eligible studies included in this meta-analysis met the following criteria: (1) reporting explicit methods for the detection of miR-375 expression in tumor tissue or blood; (2) investigating the association between miR-375 expression and survival outcome, and the end-points being overall survival (OS); (3) reporting sufficient data to estimate the hazard ratio (HR) and 95% confidence intervals (CI) according to miR-375 expression. If a study reporting the same patient cohort was included in several publications, only the most recent or complete study was selected. Studies of case reports, letters, reviews, and animal trails were excluded. The titles and abstracts of the identified articles were evaluated independently by two reviewers and the irrelevant articles were excluded. The full text of the extracted articles was carefully examined for comprehensive evaluation. The disagreements were resolved by a discussion with the third reviewer (W. Gu).

### 2.3. Data Extraction and Qualitative Assessment

The data from all eligible studies were extracted by two investigators independently, which included first authors' surname, publication year, origin of population, sample number, tumor type, follow-up period, first-line therapies, source of miRNA, miR-375 assessment methods and the cut-off definition, and HR of miR-375 expression for OS as well as 95% corresponding interval (CI) and *P* value. If a study reported the results by both univariate and multivariate analyses, the latter was selected since it considered the confounding factors and was therefore more precise.

The quality of each study was assessed independently by two researchers according to the Newcastle-Ottawa Quality Assessment Scale (NOS) [32]. For quality, scores ranged from 0 (lowest) to 9 (highest), and studies with scores of 6 or more were rated as high quality.

### 2.4. Statistical Analysis

The high or low expression of miR-375 was defined according to the cut-off values provided by the authors. HRs and their 95% CIs were combined to evaluate the association between miR-375 expression and prognosis. If the statistical variables were described in the study, we pooled them directly. Otherwise, the statistical variables were calculated from available numerical data in the articles according to the methods described by Tierney et al. [33]. To reduce reading variability, the data from Kaplan-Meier survival curves was evaluated by three independent persons as described by Engauge Digitizer version 4.1. The additional information and original data needed for the meta-analysis were acquired by contacting with the corresponding authors of eligible articles. An observed HR greater than 1 indicated a worse prognosis in patients with miR-375 downregulation. Statistical heterogeneity was assessed by visual inspection of forest plots, by performing the Chi-square test (assessing the *P* value) and calculating the *I* ^2^ statistic [34, 35]. If the *P* value was less than 0.05 and/or *I* ^2^ exceeded 50%, indicating the presence of heterogeneity, a random-effects model (the DerSimonian-Laird method) was used. Otherwise, the fixed-effects model (the Mante-Haenszel method) was used. Subgroup analysis was further performed to explore the source of heterogeneity. Publication bias was estimated by visually assessing the asymmetry of an inverted funnel plot. Furthermore, Begg's test and Egger's test were performed to provide quantitative evidence of publication bias. If a publication bias was observed, it was adjusted by the use of the Duval and Tweedie trim-and-fill method [36]. All analyses were performed using STATA vision 12.0 (Stata Corporation, College Station, TX, USA). A *P* value less than 0.05 was considered to be statistically significant except where otherwise specified.

## 3. Results

### 3.1. Study Characteristics

**Figure 1:**
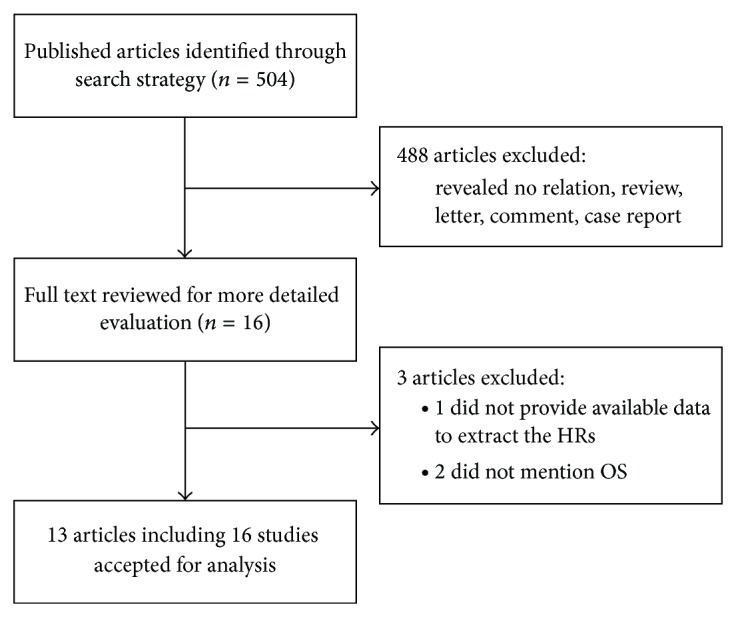
Flow chart depicting the selection of eligible studies.

According to the criteria mentioned in Materials and Methods, 504 abstracts were initially selected. However, 488 irrelevant abstracts were excluded. Sixteen full-text articles were reviewed for further evaluation. Of them, 3 were excluded because the data of HRs or OS were not available [28–30]. The remaining 13 articles contained 16 studies [12, 16–27], because one article included two independent cohort studies [16] and another article included three studies [12]. Thus, 16 studies were included in this meta-analysis, which were published between 2009 and 2014 (Figure 1). The total number of patients in all studies was 1,652, ranging from 37 to 249 patients. The category of cancers included esophageal carcinoma (8 studies), non-small-cell lung cancer (NSCLC, 3 studies), glioma, breast cancer, gastric cancer, head and neck squamous cell carcinoma (HNSCC), and pancreatic ductal adenocarcinoma (PDAC). Quantitative RT-PCR was used to detect miRNAs expression in all studies except one. The expression of miR-375 was detected in tumor tissues (11 studies) or blood samples (5 studies). The cut-off values of miR-375 varied in different studies. HRs were estimated in 8 studies and reported in the text of other studies. The major characteristics of the 16 eligible studies are listed in Table 1.

### 3.2. Qualitative Assessment

Sixteen eligible studies included in our meta-analysis were assessed for quality according to the NOS. The quality of all included studies varied from 6 to 9, with a mean of 6.6. A higher value indicated a better methodology. Therefore, all studies were included in the subsequent analysis.

### 3.3. Meta-Analysis Results

**Figure 2:**
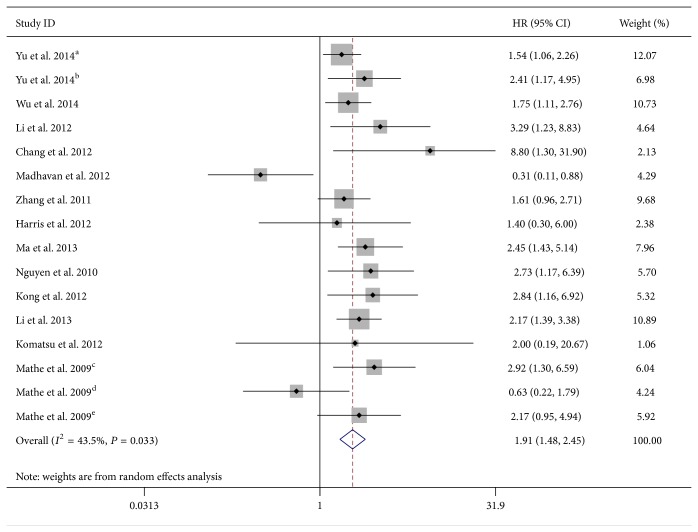
Forest plot of the relationship between lower miR-375 expression and overall survival (OS) in cancer patients with random-effects model. Different letters in superscript are represented in Table 1

As the studies evaluating OS were of obvious statistical heterogeneity (*I* ^2^ = 43.2, *P* = 0.033), we used a random-effects model to pool the HRs. The result showed that downregulated miR-375 was significantly associated with poor OS outcome in various carcinomas, with the pooled HR of 1.91 (95% CI 1.48–2.45, *P* < 0.001) (Table 2; Figure 2).

**Figure 3:**
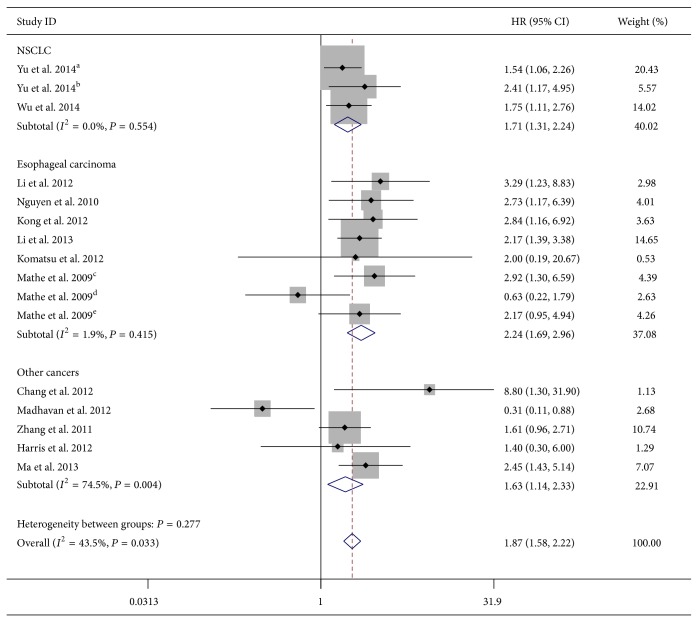
Forest plot of the relationship between lower miR-375 expression and overall survival (OS) in esophageal carcinoma and NSCLC with fixed-effects model.

Considering the heterogeneity among these studies, the effect of miR-375 expression was further evaluated by subgroup analysis. The subgroups were classified according to the main characteristics such as tumor type, source of miRNA, miRNA assay method, type of method used to obtain the HR, patient origin, and analysis type. In the subgroup of tumor type, we found the downregulation of miR-375 was significantly associated with worse OS in esophageal carcinoma (HR = 2.24, 95% CI 1.69–2.96; *P* < 0.001; fixed-effects model) and NSCLC (HR = 1.71, 95% CI 1.31–2.24; fixed-effects model), without any heterogeneity in the data (*I* ^2^ = 1.9%, *P* = 0.415; *I* ^2^ = 0.0%,*P* = 0.554, resp.) (Table 2 and Figure 3). There was only one study that evaluated the association between lower miR-375 expression and OS in HNSCC, gastric cancer, breast cancer, PDAC, and glioma, respectively, and therefore, these tumors were defined as “other cancers.” Combined data from these five studies showed that decreased miR-375 expression was not correlated with poor OS (HR = 1.59, 95% CI 0.71–3.58; random-effects model) and with significant statistical heterogeneity (*I* ^2^ = 74.5%, *P* = 0.004) (Table 2). The association between lower miR-375 expression and worse OS outcome was statistically significant in other subgroups, including HR reported in text (HR = 1.97, 95% CI 1.57–2.47, fixed-effects model; *P* = 0.346 for heterogeneity test, *I* ^2^ = 10.8%), miR-375 assay by qRT-PCR (HR = 1.88, 95% CI 1.42–2.48, random-effects model; *P* = 0.026 for heterogeneity test, *I* ^2^ = 46.2%), tissue-source of miRNA (HR = 2.16, 95% CI 1.73–2.71, fixed-effects model; *P* = 0.295 for heterogeneity test, *I* ^2^ = 15.7%), multivariate analysis (HR = 1.97, 95% CI 1.57–2.47, fixed-effects model; *P* = 0.346 for heterogeneity test, *I* ^2^ = 10.8%), Chinese patients (HR = 1.96, 95% CI 1.62–2.37, fixed-effects model; *P* = 0.295 for heterogeneity test, *I* ^2^ = 15.7%), and American patients (HR = 1.97, 95% CI 1.30–2.99, fixed-effects model; *P* = 0.180 for heterogeneity test, *I* ^2^ = 36.2%) (Table 2). The expression of miR-375 did not show prognostic impact in subgroups of HRs by data extrapolated, univariate analysis, and blood-source of miRNA. The subgroup of miR-375 detected by MISH only included one study; therefore the result related entirely to the individual study.

### 3.4. Heterogeneity Analysis

**Figure 4:**
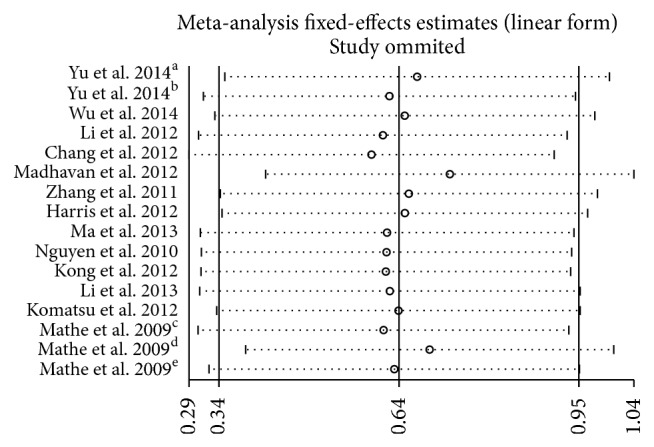
Sensitivity analysis for meta-analysis miR-375.

Sensitivity analysis was performed by sequential omission of individual studies using the fixed-effects model, demonstrating that the study by Madhavan et al. [20] apparently influenced the overall results (Figure 4). When the Galbraith plot was analyzed, one study was identified as outliers of heterogeneity [20]. By excluding this study from the analysis, similar pooled HR and significance were obtained (HR = 1.96, 95% CI 1.65–2.33, *P* < 0.001) but heterogeneity was absent (*P* = 0.395, *I* ^2^ = 5.1%). We also conducted a meta-regression to explore the potential factors responsible for the heterogeneity. As a result, all these factors including tumor type, miR-375 assay method, follow-up time, publication year, patients origin, and cut-off values did not contribute to the heterogeneity obviously.

### 3.5. Publication Bias

**Figure 5:**
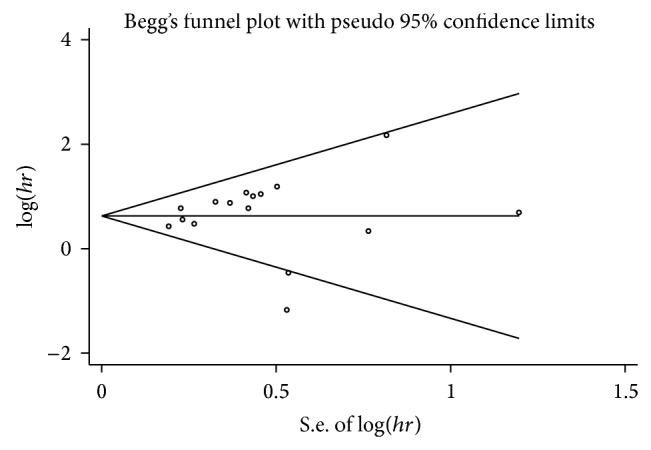
Funnel plot of lower miR-375 expression and overall survival in cancer patients.

Begg's funnel plot and Egger's test were used to evaluate the publication bias (Figure 5). The *P* values of Egger's and Begg's tests were all over 0.05 (*P* = 0.44 for Begg's test; *P* = 0.08 for Egger's test). Hence, there was no evidence for significant publication bias in the meta-analysis.

## 4. Discussion

Recently, genome-wide miRNA expression profiling studies revealed that miR-375 is widely present in various tissues and organs, and its expression is significantly aberrant in malignant tumors, such as HNSCC, NSCLC, melanoma, glioma, hepatocellular, esophageal, gastric, breast, and prostate cancer [10–16, 19, 27]. It is indubitable that miR-375 is an important cancer-related miRNA. Increasing evidence has demonstrated that miR-375 is frequently downregulated in multiple types of cancer and acts as a tumor suppressor by repressing many critical oncogenes (Table 3). In hepatocellular carcinoma, the restoration of miR-375 in cancer cells decreased cell proliferation, clonogenicity, migration, and invasion and induced G1 arrest and apoptosis [10]. Moreover, MTDH was directly regulated by miR-375 in both hepatocellular carcinoma and HNSCC [10, 37, 38]. In gastric cancer, miR-375 was frequently downregulated and inhibited gastric cancer cell proliferation via targeting Janus kinase 2 [11]. Tsukamoto et al. also found a tumor suppressive role of miR-375 in gastric cancer. The ectopic expression of miR-375 reduced cell viability and induced apoptosis by targeting PDK1 and YWHAZ [39]. Similarly, in esophageal squamous cell carcinoma miR-375 was confirmed to inhibit cell proliferation, colony formation, and metastasis in vitro and in vivo [23]. Another study also verified the tumor-suppressive effect of miR-375 in esophageal cancer cell lines by targeting PDK1 [40]. However, there were still some contradictory views requiring adequate attention. It was reported that miR-375 was significantly upregulated in tumor tissues or serum of prostate carcinoma patients [14, 41, 42], but the role of miR-375 in prostate cancer was unclear. miR-375 was also upregulated in estrogen receptor alpha- (ER*α*-) positive breast cancer cell lines, which promoted cell proliferation and induced ER*α* upregulation via RASD1, a negative regulator of ER*α* [15]. In addition, higher expression of miR-375 was reported to result in progression of invasive lobular breast carcinoma [43]. Recently, miR-375 was found to participate in the process of epithelial-to-mesenchymal transition (EMT). It could partly reverse EMT-like properties in MCF-7 cells [44], which suggested its promoting role in tumor metastasis. Therefore, whether miR-375 acts as a tumor suppressor or an oncogene is unclear. Although plenty of studies focused on the potential application of miR-375 as a prognostic biomarker, the prognostic value of miR-375 expression in cancer patients is still controversial.

In the present study, we carried out a meta-analysis to evaluate the prognostic value of miR-375 by combining 16 studies with 1,652 patients. Our results demonstrated that lower miR-375 expression was associated with poor survival in patients with various carcinomas (HR = 1.91 95% CI 1.48–2.54, *P* < 0.001, random-effects model), indicating that miR-375 may serve as a positive prognostic marker for solid tumors. In the subgroup analyses, the association between lower miR-375 expression and worse OS was statistically significant in most subgroups, especially in esophageal carcinoma (HR = 2.24, 95% CI 1.69–2.96; *P* < 0.001; fixed-effects model) and NSCLC (HR = 1.71, 95% CI 1.31–2.24; fixed-effects model). Recently, there were only two studies exploring the relationship between miR-375 expression and the survival of cancer patients. Consistent with our results, they also indicated low miR-375 expression was significantly associated with poor OS of patients with esophageal carcinoma. However, the results of these two studies are not credible enough because they did not eliminate interference factors from their meta-analyses, including miR-375 expression in pericarcinoma tissues of the same patient cohort, which may possibly come to a conclusion with deviation.

In sensitivity analysis, the study of Madhavan et al. was identified as an outlier of heterogeneity. It indicated a positive correlation between upregulated miR-375 expression and poor OS in metastatic breast cancer (MBC) patients (HR = 0.31, 95% CI 0.11–0.88) [20], which may be attributed to mesenchymal-to-epithelial transition (MET), a process essential for successful colonization and establishment of metastasis induced by high miR-375 expression [44]. The significant prognostic effect of high miR-375 expression in this study with obvious heterogeneity possibly led to the negative statistical results in some subgroup analyses, including data extrapolated, univariate analysis, and blood-source of miRNA: in these subgroups, there was no association between lower expression of miR-375 and poor prognosis.

Although the predictive value of miR-375 was statistically proved by the meta-analysis in this study, it should be carefully comprehend for the following reasons. First, four studies which explored the association between miR-375 expression and prognosis were not included in the meta-analysis because they did not provide available data of HRs or OS, and three of them showed insignificant or even opposite results. These studies might influence the reliability of our results. Secondly, due to the lack of uniform cut-off value in miR-375 expression, the cut-off values applied by different researchers deviated from the actual value more or less, which may affect the validity of miR-375 as a predictive factor in cancer prognosis. Thirdly, several HRs were calculated based on the data extracted from the survival curve, bringing minor deviations. Fourthly, the statistical heterogeneity was not obvious in this meta-analysis, but the clinical and methodological heterogeneity existed in the baseline demographic characteristics, including population, tumor types and stages, the cut-off value of miR-375 expression, and duration of follow-up. Finally, although no significant publication bias was detected in this meta-analysis, the results still need to be verified by a large number of publications.

In conclusion, this meta-analysis summarized the global researches on the relationship of aberrant miR-375 expression and the prognosis of patients with cancer and clarified that downregulation of miR-375 is significantly associated with poor survival in patients with various types of carcinoma, especially in esophageal carcinoma and NSCLC. In view of the limitation of the current analysis, it should be cautious to appreciate the conclusion, and further prospective multicenter studies designed adequately with larger sample size are needed to verify the association between miR-375 and cancer prognosis as well as the efficiency of therapies.

## Figures and Tables

**Table 1:** Main characteristics of all studies included in the meta-analysis.

Study	Origin of population	Tumor type	Age (years, *n*)	Gender (M/F)	Sample number	Stage	Follow-up (months)	Source of miRNA	miR-375 assay	Cut-off definition	Multivariate analysis	Survival analysis	HR
Yu et al. 2014^a^ [16]	China	NSCLC	<60, 96; ≥60, 68	86/78	164	I–IV	Median 24 (rang: 0–56)	Blood	qRT-PCR	Median	Yes	OS	Report
Yu et al. 2014^b^ [16]	China	NSCLC	NR	NR	53	I–IV	Median 24 (rang: 0–56)	Blood	qRT-PCR	Median	Yes	OS	Report
Wu et al. 2014 [17]	China	ESCC	Mean 61.4 (33–81)	115/79	194	I–IV	Median 31.3	Blood	qRT-PCR	Mean	Yes	OS	Report
Li et al. 2012 [18]	China	NSCLC	Median 61 (37–75)	70/26	96	I–III	Median 30 (rang: 8–69)	Tissue	qRT-PCR	Mean	Yes	OS	Report
Chang et al. 2012 [19]	China	Glioma	Median 42 (12–71)	76/52	128	I–IV	60	Tissue	qRT-PCR	Median	Yes	OS	Report
Madhavan et al. 2012 [20]	Germany	Breast	NR	0/164	164	IV	NR	Blood	qRT-PCR	Lower quartile	No	OS, PFS	DE
Zhang et al. 2011 [26]	China	Gastric	30–83	43/22	65	I–IV	Over 60	Tissue	qRT-PCR	2-fold	No	OS	DE
Harris et al. 2012 [27]	USA	HNSCC	62.2 ± 11.9	85/38	123	I–IV	NR	Tissue	qRT-PCR	Lower quartile	Yes	OS	Report
Ma et al. 2013 [21]	China	PDAC	NR	44/34	78	I–IV	NR	Tissue	qRT-PCR	Mean	Yes	OS	Report
Nguyen et al. 2010 [22]	USA, Canada	EADC	32–80	35/23	58	I–IV	Median 72	Tissue	qRT-PCR	Median	Yes	OS	Report
Kong et al. 2012 [23]	China	ESCC	Mean 66	43/17	60	I–IV	Over 60	Tissue	qRT-PCR	Normal	No	OS, DFS	DE
Li et al. 2013 [24]	China	ESCC	≤60, 105; >60, 144	136/113	249	I–IV	Over 60	Tissue	MISH	Normal	No	OS	DE
Komatsu et al. 2012 [25]	Japan	ESCC	Median 65	44/6	50	0–IV	36	Blood	qRT-PCR	Median	No	OS	DE
Mathe et al. 2009^c^ [12]	USA, Canada	EADC	NR	NR	63	I–IV	Median 60	Tissue	qRT-PCR	Median	No	OS	DE
Mathe et al. 2009^d^ [12]	USA, Canada	EADC	NR	NR	37	I–IV	Median 60	Tissue	qRT-PCR	Median	No	OS	DE
Mathe et al. 2009^e^ [12]	USA, Japan	ESCC	<62, 28; ≥62, 42	52/18	70	I–IV	Median 60	Tissue	qRT-PCR	Median	No	OS	DE

NSCLC: non-small cell lung cancer; ESCC: esophageal squamous cell carcinoma; HNSCC: head and neck squamous cell carcinoma; EADC: esophageal adenocarcinoma; qRT-PCR: quantitative real-time PCR; MISH: miRNA in situ hybridization; OS: overall survival; DFS: disease-free survival; PFS: progression-free survival; HR: hazard ratio; DE: data extrapolated; NR: not reported.

^a^Study by Yu et al. [16] that evaluated the association between miR-375 expression and OS in a discovery cohort of 164 NSCLC patients.

^b^Study by Yu et al. [16] that evaluated the association between miR-375 expression and OS in a validation cohort of 53 NSCLC patients.

^c^Study by Mathe et al. [12] that evaluated the association between miR-375 expression and OS in a validation cohort of 63 EADC patients with Barrett's.

^d^Study by Mathe et al. [12] that evaluated the association between miR-375 expression and OS in a validation cohort of 37 EADC patients without Barrett's.

^e^Study by Mathe et al. [12] that evaluated the association between miR-375 expression and OS in a validation cohort of 70 ESCC patients.

**Table 2:** The pooled associations between different situations of miR-375 expression and the prognosis of patients with solid tumors.

Outcome subgroup	Number of patients	Number of studies	HR (95% CI)	*P* value	Heterogeneity	
					*I* ^2^	*P*
Overall effect	1652	16	1.91 (1.48–2.45)^b^	<0.001∗	43.2	0.033
Tumor type						
Esophageal carcinoma	781	8	2.24 (1.69–2.96)^a^	<0.001∗	1.9	0.415
NSCLC	313	3	1.71 (1.31–2.24)^a^	<0.001∗	0.0	0.554
Other cancers	558	5	1.59 (0.71–3.58)^b^	0.26	74.5	0.004
miR-375 assay method						
QRT-PCR	1403	15	1.88 (1.42–2.48)^b^	<0.001∗	46.2	0.026
MISH	249	1	1.99 (1.28–3.10)	0.002∗		
HR obtain method						
Reported in text	894	8	1.97 (1.57–2.47)^a^	<0.001∗	10.8	0.346
Data extrapolated	758	8	1.58 (0.99–2.51)^b^	0.054	61.6	0.011
Analysis type						
Multivariate	894	8	1.97 (1.57–2.47)^a^	<0.001∗	10.8	0.346
Univariate	758	8	1.58 (0.99–2.51)^b^	0.054	61.6	0.011
Source of miRNA						
Tissue	1027	11	2.16 (1.73–2.71)^a^	<0.001∗	15.7	0.295
Blood	625	5	1.40 (0.84–2.35)^b^	0.197	63.5	0.027
Patient origin						
China	1087	9	1.96 (1.62–2.37)^a^	<0.001∗	5.7	0.388
USA	351	5	1.97 (1.30–2.99)^a^	=0.001∗	36.2	0.180
Other countries	214	2	0.58 (0.10–3.24)^b^	0.534	50.7	0.154

NSCLC: non-small-cell lung cancer; qRT-PCR: quantitative real-time PCR; MISH: miRNA in situ hybridization; HR: hazard ratio; CI: confidence intervals.

∗The difference was statistically significant.

^a^Fixed-effects model.

^b^Random-effects model.

**Table 3:** The dysregulated expression and target genes or pathways of miR-375 in cancer.

Type of cancer	miR-375 expression	Validated target genes or pathways	Reference
HNSCC	Down	MTDH, LDHB, IGF1R	[37, 38, 45, 46]
ESCC	Down	PDK1, IGF1R	[23, 40]
Gastric cancer	Down	JAK2-STAT3, PDK1, YWHAZ, ERBB2	[11, 39, 47–49]
HCC	Down	AEG-1, ATG7, YAP1	[10, 50–52]
Lung cancer	Down	YAP1, CLDN1	[53, 54]
Pancreatic cancer	Down	PDK1	[55, 56]
Colorectal cancer	Down	PIK3CA	[57, 58]
Cervical cancer	Down	SP1	[59, 60]
Breast cancer	Up	RASD1, IGF1R	[15, 61]
Prostate cancer	Up	Sec23A	[14]

HNSCC: head and neck squamous cell carcinomas; ESCC: esophageal squamous cell carcinoma; HCC: hepatocellular carcinoma.
